# Methamphetamine enhances neural activation during anticipation of loss in the monetary incentive delay task

**DOI:** 10.1093/texcom/tgad014

**Published:** 2023-07-20

**Authors:** Hanna Molla, Sarah Keedy, Joseph DeBrosse, Harriet de Wit

**Affiliations:** Department of Psychiatry and Behavioral Neuroscience, University of Chicago, Chicago, IL 60637, USA; Department of Psychiatry and Behavioral Neuroscience, University of Chicago, Chicago, IL 60637, USA; Department of Psychiatry and Behavioral Neuroscience, University of Chicago, Chicago, IL 60637, USA; Department of Psychiatry and Behavioral Neuroscience, University of Chicago, Chicago, IL 60637, USA

**Keywords:** fMRI, methamphetamine, reward, stimulant, ventral striatum

## Abstract

Stimulants like methamphetamine (MA) affect motivated behaviors via actions on circuits mediating mood, attention, and reward. Few studies examined the effects of single doses of stimulants on reward circuits during anticipation and receipt of rewards and losses. Here, we examined the effects of MA (20 mg) or placebo in a within-subject, double-blind study with healthy adults (*n* = 43). During 2 fMRI sessions, participants completed the monetary incentive delay task. Primary outcome measures were BOLD activation in selected regions of interest during anticipation and receipt of monetary rewards and losses. Secondary analyses included behavioral measures, whole brain analysis, and arterial spin labeling. MA produced its expected behavioral effects and increased neural activation in the ventral striatum and anterior insula during anticipation of monetary loss versus non-loss. MA did not affect activation during anticipation of gains, or during receipt of wins or losses. MA significantly reduced cerebral blood flow in the striatum and insula. The present finding that a stimulant enhances the responses of striatal and insular regions to upcoming loss suggests that this system may be sensitive to the salience of upcoming events. The finding adds to a complex body of evidence regarding the effects of stimulant drugs on neural processes during motivated behaviors.

## Introduction

Brain processing of reward and loss plays a key role in how we learn and make everyday decisions, guiding approach toward positive stimuli and avoidance of negative stimuli (Lutz and Widmer 2014). Drugs that act on brain reward circuits, such as stimulants, can be used to investigate the regions involved in various components of reward processing. For example, amphetamine-like drugs activate neural circuits involved in reward processing by enhancing monoaminergic transmission, especially dopamine and norepinephrine (Rothman et al. 2001; Cruickshank and Dyer 2009). In addition to their effects on reward circuits, these drugs also affect mood states and other cognitive processes or attentional processes, perhaps through different brain circuits (e.g. salience and executive networks; Wilson et al. 2018). One way to study the mechanisms by which stimulants alter motivated behavior is by studying their effects on regional brain function during tasks that involve anticipation and receipt of cues signaling both reward and loss. Here, we used the monetary incentive delay task (MID; Knutson et al. 2004) to investigate the effects of a stimulant drug on this task.

The MID has been widely used to investigate cortical and subcortical regions mediating many aspects of reward learning, including attention, anticipation versus receipt of outcomes, reward versus loss, and learning (Knutson and Greer 2008; Lutz and Widmer 2014; Luijten et al. 2017; Dugré et al. 2018; Oldham et al. 2018; Wilson et al. 2018; Jauhar et al. 2021). In one meta-analysis, Oldham et al. (2018) concluded that anticipating both rewards and losses recruits the striatum, insula, amygdala, and thalamus, whereas specific reward outcomes involved mainly the orbitofrontal/ventromedial prefrontal regions. Other studies have implicated behavioral and neural processes indirectly related to reward. For example, Zink et al. (2004) found that involvement of the striatum in reward processing depended more on the saliency associated with reward, rather than value or hedonic feelings related to the outcome. Using a procedure that separated activation induced from motivation versus reward value, Miller et al. (2014) found that motivation altered activity in the caudate and putamen, whereas expected reward altered activity in the nucleus accumbens. Despite this complexity, examining the effects of a stimulant drug on the MID task may shed light on the neural processes underlying a reward.

Methamphetamine (MA) acts on relevant brain networks through its ability to increase dopamine, norepinephrine, and to a lesser extent serotonergic, transmission (Rothman et al. 2001). Animal studies revealed that acute exposure to MA in rodents increases locomotor activity and elicits higher levels of extracellular dopamine in the striatum including the caudate, putamen, and nucleus accumbens (Zhang et al. 2001; Izawa et al. 2006). One early functional magnetic resonance imaging (fMRI) study with healthy volunteers showed that intravenous administration of MA activated reward and attention-related regions including the orbitofrontal cortex (OFC) and ventral striatum (Völlm et al. 2004). In another study, a single oral dose of MA increased functional connectivity between the nucleus accumbens and OFC, and between the putamen and inferior frontal cortex, during the resting state conditions (Weafer et al. 2020). Another study (Bernacer et al. 2013) examined the effects of MA on neural activity during prediction errors, which are thought to be a fundamental aspect of learning. Prediction error signals arise from discrepancies between expected and received outcomes of rewarding or aversive stimuli, leading to increases and decreases in the firing of dopamine neurons in the striatum (O'Doherty 2004). Single doses of MA decreased neural activation in the nucleus accumbens during reward prediction error signals and in the ventromedial prefrontal cortex (vmPFC) during incentive value processing. A recent study from our lab using a monetary reward task found that a single dose of MA decreased nucleus accumbens activation during receipt of monetary reward in contrast to loss, and this effect was primarily due to an increased effect of the drug on receipt of losses (Crane et al. 2023). These studies collectively provide insights into how MA affects reward and attention-related regions. They suggest that due to its complex pharmacological profile, MA alters neural responses to rewards, influences the evaluation of expected and received outcomes, and may have differential effects on the processing of rewarding and aversive stimuli. We can gain further insights into how this drug modulates regions involved in reward and attentional processing using controlled administration of the drug in a well-characterized task.

Several previous studies have examined the acute effects of stimulant drugs on brain activity during the MID. Knutson et al. (2004) studied the effects of d-amphetamine (about 20 mg oral) in 8 healthy adults. Under placebo conditions, they found that a cue signaling upcoming reward altered activation in the ventral striatum and increased peak activation during the anticipation of loss. d-amphetamine decreased the peak magnitude of ventral striatum activation during anticipation of reward (no effect on receipt of reward). The authors speculated that amphetamine reduced the signal during anticipation of gain because of the drug’s enhancement of baseline (tonic) dopaminergic transmission (Schmitz et al. 2001). Similar results were reported by Schouw et al. (2013) who found that methylphenidate (35 mg oral) reduced the striatal activation during reward anticipation in 8 healthy adults. One other study (Funayama et al. 2014), however, found that the stimulant-like drug modafinil increased BOLD signal in the nucleus accumbens in anticipation of high magnitude rewards, without altering activation during anticipation of losses. The differences in findings could be due to procedural differences or to differences between the drugs.

In this study, we used the MID task (Knutson et al. 2004) to examine the effect of MA (20 mg) on neural responses to the anticipation and receipt of monetary reward and loss in 48 healthy adults. The study extended previous studies by testing a larger sample of men and women. To analyze the BOLD data, we used both a focal analysis of a priori regions previously identified as affected by stimulants and an exploratory whole brain analysis to assess effects of the drug on global activation.

## Materials and methods

### Design overview

In a within-subject double-blind study, healthy men and women received MA (20 mg) or placebo in randomized order across 2 sessions. At the time of peak drug effect, participants underwent an fMRI scan and completed the MID task. Subjective drug and mood ratings and cardiovascular measures were obtained at regular intervals during the sessions.

### Participants

Healthy male and female volunteers (*n* = 48) between the ages of 18 and 35 participated in the study. They were recruited via flyers and advertisements on social media. Participants underwent in-person screening including a physical exam, electrocardiogram, psychiatric screening interview, medical, and drug use history assessments. Inclusion criteria included being right-handed, normal electrocardiogram, BMI between 19 and 26, fluent in English, and at least a high school education. Exclusion criteria were history of psychosis, severe post-traumatic stress disorder, depression, current suicidal ideation, prescription medication use (other than birth control), contraindications for MRI scan, pregnancy (verified by pregnancy tests on sessions days), history of cardiovascular disease, or consuming more than 4 alcohol or caffeinated beverages a day. Females who were not on hormonal birth control attended sessions during the follicular phase of their menstrual cycle (White et al. 2002).

### Procedure

Participants provided written, informed consent and attended an orientation session, where they practiced the tasks. The protocol was approved by the Institutional Review Board of the University of Chicago. To minimize drug expectancies, participants were informed they would ingest capsules that could contain a placebo, a sedative, or a stimulant. They were instructed to fast for at least 8 h prior to the start of the sessions, abstain from recreational drug use for at least 48 h, and alcohol for at least 24 h before the sessions. In addition to the 2 fMRI sessions presented here, participants also attended 2 sessions in which they received MA and placebo in a naturalistic non-imaging laboratory setting. The results of the 2 non-imaging sessions are not presented here. Participants also completed some drug rating scales and tasks that are not presented here.

The 2 4-h sessions were conducted from 9:00 AM to 1:00 PM, separated by at least 72 h. Upon arrival in the laboratory, drug abstention was verified by urine screening (CLIAwaived Instant Drug Test Cup) and breath alcohol test (Alcosensor III, Intoximeters, St. Louis, MO), and women were tested for pregnancy. Participants completed the baseline ratings on questionnaires (see below) and provided cardiovascular measures. Then at 9:30 AM, MA (20 mg; Desoxyn tablets placed in size 00 opaque capsules with dextrose filler) or placebo (dextrose) was administered under randomized, double-blind conditions. After taking the capsule, participants were given a granola bar as a standardized breakfast and relaxed in a comfortable room for 1 h. At 10:30 AM, they completed subjective and cardiovascular measures and were taken to the imaging center for the fMRI scan (see below). After the scan, subjective and cardiovascular measures were obtained at regular intervals. At 1:00 PM, participants were discharged after confirming cardiovascular measures were within 20% of baseline. After completing all sessions, participants were debriefed and compensated.

### Measures

1. **Drug effects questionnaire** (DEQ; Fischman and Foltin 1991; Morean et al. 2013). The DEQ consists of questions on a visual analog scale about the subjective effects of drugs. For the present analyses, we examined the extent to which they felt any drug effect, rated on a 100 mm line, from “Not at all” (0) to “Very much” (100). They completed this before taking the capsule and 30, 50, 120, 135, 180, 210 min after drug administration.
2. **MID task** (Knutson et al. 2000; Wu et al. 2014). This task was designed to evoke neural responses during both anticipation and receipt of monetary reward and loss. At the beginning of each task trial, participants were presented with 1 of 6 cues for 2,000 ms, indicating either gain (circle) or loss (square) of a varying magnitude (± $5.00, ± $1.00) and neutral cues (± $0.00). Actual values were displayed underneath the cues. Following cue presentation, a fixation cross appeared on the screen for 2,000–2,500 ms, which provided the window for the anticipation phase. The fixation cross was followed by the presentation of a triangle target (150–500 ms), and subjects were instructed to press a key before this target disappeared. If they successfully responded to the target, they won the specified amount of money (for the gain trials) or avoided losing that amount (for the loss trials). Participants were then informed of the outcome of the trial, which was the “feedback” stimulus (2,000 ms) that initiated the feedback period. Each trial was separated by a varying intertrial interval of 2,000–6,000 ms. Each of the 6 trial types (valence [2] × magnitude [3]) were completed 15 times, in randomized order, totaling 90 trials. The duration of the triangle target was adapted such that the maximum rate at which participants could successfully hit the target was 66%. To confirm task engagement, we assessed responses to the trials and excluded 5 outliers with hit rates of less than 44% on at least 1 of the scans. We also recorded the hit reaction times for all condition trials.
3. **Cardiovascular measures.** Blood pressure and heart rate were monitored using portable blood pressure cuffs (Omron BP791IT, Omron Healthcare). Cardiovascular measures were taken before drug administration, and at 30–60-min intervals throughout the session for a total of 6 timepoints. We calculated the peak change from baseline during each session and performed 2-tailed paired *t*-tests to compare between MA and placebo conditions.

### fMRI data acquisition

Functional MRI data were collected at the University of Chicago MRIRC using a 3 T Philips Achieva scanner with a 32 receiver channel head coil and a gradient-echo echo-planar imaging sequence with the following acquisition parameters: TR = 2,000 ms; TE = 28; 39 3 mm thick axial slices aligned to the AC-PC line, 0.6 mm slice gap; 20 × 20 cm field-of-view; SENSE factor = 2, Flip angle = 77°. Four initial volumes were acquired and discarded just prior to task start, to allow for T1 equilibrium effects. A high resolution T1-weighted image (MPRAGE sequence) was also acquired to assess for incidental findings, and for alignment and spatial standardization of the functional data. Motion was minimized with foam packing around the head. Stimuli were viewed via projection onto a mirror mounted on the head coil. We also acquired arterial spin labeling (ASL) images to assess perfusion changes. Parameters included: TR = 4,692 ms; TE = 13 ms; Flip angle = 90 °; labeling time = 1,800 ms; post label delay = 1,800 ms. A total of 35 pairs of ASL volumes were acquired.

### Participant-level functional image processing

In addition to the BOLD data, we also collected reversed phase encode blips to correct for geometric distortion. From the pairs of images, we estimated the susceptibility-induced off-resonance field using a similar method to that described in Andersson et al. (2003), as implemented in FSL (Smith et al. 2004). The 2 images were combined into a single corrected one, and they collectively corrected the fMRI time series. Following this, image preprocessing was carried out using AFNI (Cox 1996). Preprocessing steps included alignment of the time series to the volume with the minimum outlier fraction, spatial registration of the aligned time series data to the anatomical scan, anatomical scan warping to MNIspace and warp applied to functional data, spatially smoothed with a 5 mm FWHM Gaussian kernel, and intensity normalization. Volumes exceeding a motion-related displacement of >3 mm were excluded from the analysis. Participants with greater than 50% of TRs censored during at least 1 scan were excluded for excessive motion. Voxelwise neural activation was then estimated for each participant using a multiple linear regression analysis via AFNI 3dDeconvolve. De-meaned and derivatives of motion parameters and white matter signal were estimated and included as covariates of non-interest. First-level analyses included contrasts for anticipation and feedback phases (Knutson et al. 2004; Wu et al. 2014). Contrasts for the anticipation phase included: (i) gain (+$1.00, +$5.00) versus non-gain (+$0.00) anticipation, (ii) loss (−$1.00,−$5.00) versus non-loss (−$0.00) anticipation, and (iii) gain (+$1.00, +$5.00) versus loss (−$1.00,−$5.00) anticipation. Contrasts for the feedback (reward outcome) phase included: (a) hit (+$1.00, +$5.00) versus miss (+$0.00) gain outcomes, and (b) hit (−$0.00) versus miss (−$1.00,−$5.00) loss outcomes.

### Statistical analysis

Analyses were conducted as within-subject comparisons, contrasting MA and placebo sessions. A priori regions of interest (ROI) for the anticipation phase included the ventral striatum, thalamus, anterior insula, and caudate. Masks for each ROI were created using spheres with a radius of 8 mm at coordinates reported from Oldham et al. (2018). For the anticipation phase, mean *t* values for each contrast (gain vs. non-gain, loss vs. non-loss, gain vs. loss, and gain/loss vs. non-gain/non-loss) were extracted from placebo and MA sessions for each ROI. For the feedback phase, ROI’s included the ventral striatum, amygdala, OFC/vmPFC, and posterior cingulate cortex (PCC). Mean *t* values were extracted for the following contrasts: gain hit versus miss and loss hit versus miss. Repeated measures analyses of variance (ANOVAs) were conducted with drug (placebo, MA) as the within-subject factor of interest, and sex and order (placebo-MA, MA-placebo) as between-subject factors of non-interest (SPSS Version 25) for all contrasts in both anticipation and feedback phases. Adjustments for multiple comparisons were made using Bonferroni-corrected thresholds of significance (*P* < 0.013).

Exploratory whole-brain analyses were conducted using AFNI’s 3dttest++, implementing a paired *t*-test comparing MA versus placebo activation (for all anticipation and reward outcome contrasts) on a voxelwise basis, using age, sex, and motion as covariates. Clusters of voxels were determined to be significant at a familywise error correction threshold of *P* < 0.05, where at least 9 contiguous voxels each met *P* < 0.001. This threshold was determined using 3dClustSim that ran simulations incorporating mean autocorrelation values from all included subjects’ preprocessing estimates, a gray matter mask, and the same 3 × 3 × 3 geometry as our data.

ASL data underwent preprocessing using FSL BASIL toolbox for cerebral blood flow (CBF) map quantification (Chappell et al. 2008). This process involved motion correction, co-registration, quantification of CBF maps according to the method used by Alsop et al. (2015), and warping into MNI space. We assessed for CBF differences between placebo and drug sessions only where significant BOLD changes were also found, extracting CBF values using the same ROI masks, and comparing the CBF values using repeated measures ANOVA (SPSS Version 25) with age as a covariate. We excluded 1 participant from ASL analysis due to missing data from 1 session.

## Results

### Participants

Five participants were excluded from analysis due to poor task performance and 1 participant was excluded due to excessive movement. Participants in the final analysis included 23 men and 20 women in their mid-20s with an average BMI of 24.1. Out of the 43 participants, 10 reported ever having used a stimulant drug for nonmedical purposes. These 10 participants had used the drug a mean of 2.8 (±2.8 SD) times in their lives (Table 1).

**Table 1:** Participant demographics.

	*n* (%) or mean (SD)
Sex (M/F)	23/20
Age	25.1 (4.3)
Race/Ethnicity	
*Asian*	7 (16%)
*Black*	3 (7%)
*Hispanic/Latino*	5 (12%)
*White*	25 (58%)
*Other/More than one*	3 (7%)
BMI	24.1 (2.7)
Education (in years)	15.6 (1.5)
Current drug use	
*Caffeine drinks per day*	1.2 (1.1)
*Cigarettes per day*	0.2 (0.8)
*Alcoholic drinks per week*	1.4 (1.1)
*Cannabis use in past 30 days*	4.8 (7.4)
Lifetime drug use (times ever used)	
*Stimulants*	0.7 (1.8)

### Subjective and physiological effects of the drug

**Fig. 1:**
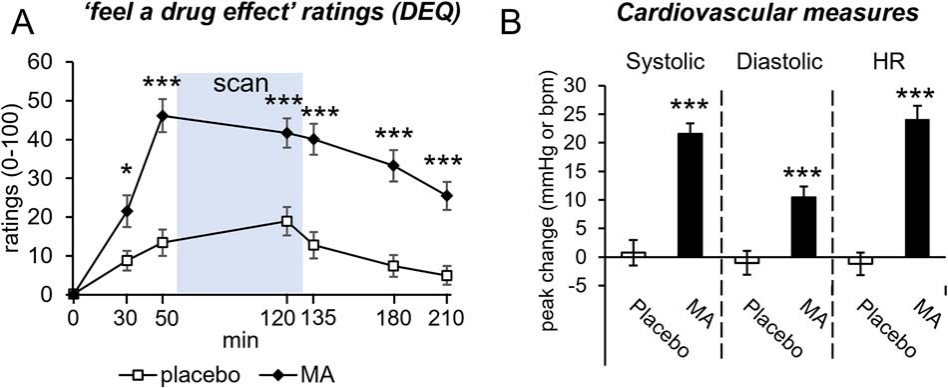
A) Mean (SEM) ratings for “feeling a drug effect” at specified intervals after administration of MA (20 mg) and placebo. Shaded area represents time spent in the scanner. Drug × timepoint interaction, *F*(6,240) = 9.9. B) Mean (SEM) peak change from baseline scores of systolic and diastolic blood pressure (in mmHg), and heart rate (HR; in beats per minute [bpm]) during MA (black bars) and placebo (white bars) sessions. *P* < 0.05^*^, *P* < 0.0005^*^^*^^*^.

MA significantly increased ratings for “feeling a drug effect” (DEQ) relative to placebo at each time point post-capsule administration, including during the period while participants were completing the MID task (Fig. 1A; repeated measures ANOVA, drug × timepoint interaction, *F*(6,240) = 9.9, *P* < 0.05^*^, *P* < 0.0005^*^^*^^*^). MA significantly increased peak changes from baseline for all cardiovascular measures including systolic (*P* < 0.0001) and diastolic (*P* < 0.0005) blood pressure, and heart rate (*P* < 0.0001), relative to placebo (Fig. 1B).

### Imaging data

#### Activation during MID on placebo sessions

**Fig. 2:**
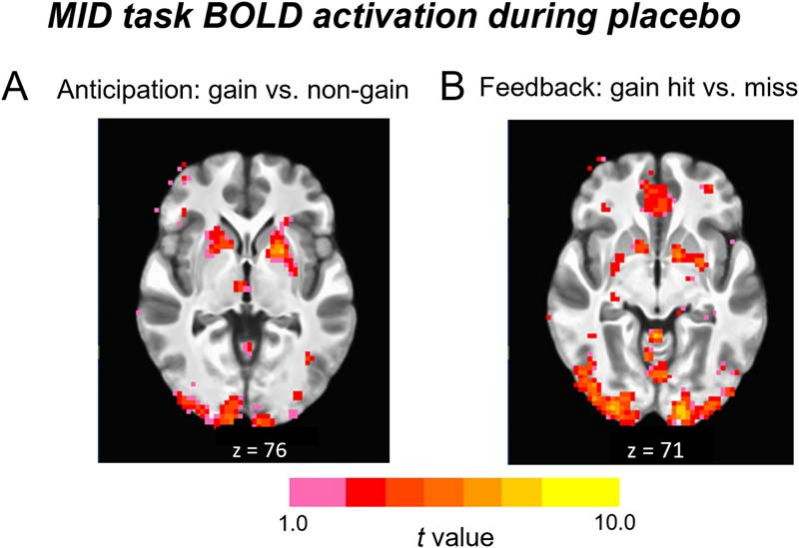
Axial image showing BOLD activation during placebo on A) the anticipation phase of the MID task in the gain (+$1.00 and +$5.00) versus non-gain (+$0) contrast, and B) during the MID feedback phase in the gain hit (+$1.00, +$5.00) versus miss (+$0.00) contrast.

To confirm that the MID task produced activation in expected regions (Knutson and Greer 2008; Wu et al. 2014), we assessed whole brain activation on placebo sessions for both anticipation and feedback phases. We detected increases in putamen and ventral striatum during anticipation of gaining the monetary reward relative to a neutral cue, as expected. During feedback, we detected increases in the mesial prefrontal cortex during receipt of monetary reward compared to neutral condition (Fig. 2).

#### Effects of MA on reward-related ROI’s during the MID anticipation phase

There were no significant main effects of sex or order, or significant interactions of drug × sex (1 drug × order interaction, *see below*).

*Gain* (+$1, +$5) versus *non-gain* (*+*$0) *anticipation*: In this comparison, MA did not significantly alter BOLD activation in any of the reward-related ROI’s: ventral striatum, thalamus, anterior insula, or caudate (Supplementary Table 1).

**Fig. 3:**
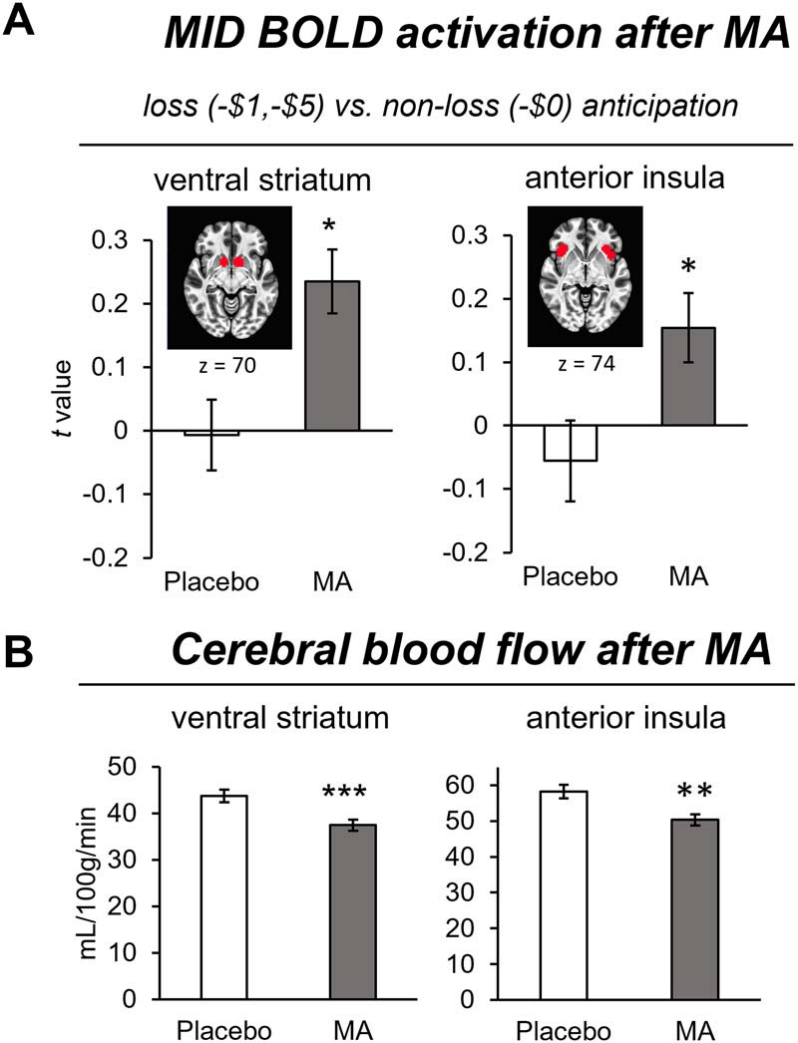
A) Mean (SEM) *t* values for BOLD activation during anticipation of loss in the ventral striatum and anterior insula after administration of MA (gray bars) and placebo (white bars). *P* < 0.05^*^. B) Mean (SEM) CBF values for ventral striatum and anterior insula regions after administration of MA (gray bars) and placebo (white bars). *P* < 0.005^*^^*^, *P* < 0.0005^*^^*^^*^.

*Loss* (−$1.00, −$5.00) versus *non-loss* (*−*$0) *anticipation*: In this comparison, compared to placebo, MA increased BOLD activation of the ventral striatum (main effect drug, *F*(1,39) = 8.1, *P* = 0.007) and anterior insula (main effect drug, *F*(1,39) =8.2, *P* = 0.007; drug^*^order interaction, *F*(1,39) = 7.4, *P* = 0.01), during anticipation of loss versus non-loss (Fig. 3A). Differences in activation between drug conditions were not observed in the thalamus or caudate.

*Gain* (+$1.00, +$5.00) versus *loss* (−$1.00, −$5.00) *anticipation*: MA did not significantly alter activation relative to placebo during anticipation of gains versus losses in the ventral striatum, thalamus, anterior insula, or caudate.

#### Effects of MA on reward-related ROI’s during the MID feedback (reward outcome) phase

MA did not significantly alter BOLD activation in reward-related ROIs including the ventral striatum, amygdala, OFC/vmPFC, and PCC for either gain (hit vs. miss) or loss (hit vs. miss) reward outcome contrasts (Supplementary Table 1).

#### Exploratory whole brain analyses

MA did not significantly alter any clusters in the whole brain analyses during either anticipation or feedback phases, using a threshold of *P* < 0.001, α = 0.05.

#### MID task reaction times

MA decreased reaction times during hit trials, relative to placebo (repeated measures ANOVA with drug condition and cue value as within-subject factors; main effect of drug, *F*(1,33) = 6.7, *P* = 0.01). Further analyses showed that participants responded faster to cues associated with reward and loss (±$1, ±$5) relative to neutral cues (±$0; main effect of cue value (*F*(5,165) = 21.0, *P* < 0.001; all *P*-values < 0.01; Table 2). Hit reaction time (placebo subtracted from drug) was not significantly related to ventral striatal BOLD activation (placebo subtracted from drug) during anticipation of loss versus non-loss (Pearson correlation, *r* = 0.166, *P* = 0.4).

**Table 2:** Mean (±SEM) reaction time for each cue condition.

**Hit reaction time (ms)**	**Placebo** **mean (±SEM)**	**MA** **mean (±SEM)**
+$0.00	232.8 (0.005)	218.9 (0.003)^*^
+$1.00^#^	217.2 (0.003)	206.3 (0.004)^*^
+$5.00^#^	210.6 (0.004)	204.9 (0.003)^*^
−$0.00	224.8 (0.005)	213.9 (0.003)^*^
−$1.00^#^	216.2 (0.003)	211.4 (0.004)^*^
−$5.00^#^	211.0 (0.003)	206.4 (0.003)^*^

Main effect of drug, MA versus placebo (*P* < 0.05^*^). Main effect of cue-value, reward or loss versus neutral cues (*P* < 0.05^#^).

#### ASL CBF

We examined changes in CBF within the ventral striatum and the anterior insula, regions where the drug effect on BOLD signal was significant, between MA and placebo conditions. We conducted this assessment to examine the influence of MA on blood flow independent of neural activity evoked by the task. We found MA significantly decreased CBF in the ventral striatum (*F*(1, 27) = 17.8; *P* < 0.0002) and anterior insula (*F*(1, 27) = 12.3; *P* < 0.002), relative to placebo (Fig. 3B). Age was not a significant covariate (*P* > 0.1). The MA-induced decrease in ventral striatal blood flow was not related to the MA-induced increase in striatal BOLD activation during anticipation of loss versus non-loss (*r* = −0.012, *P* = 0.9).

## Discussion

This study examined the effects of MA on neural activation during performance on a MID task, in 43 healthy adults. Our primary finding was that, using ROI analysis, the single oral dose of MA (20 mg) increased activation in the ventral striatum and anterior insula relative to placebo during anticipation of monetary loss (vs. non-loss), but not during either anticipation of gain (vs. non-gain) or during feedback for either gains or losses. In exploratory whole brain analyses, the drug did not significantly affect regional activation during either anticipation or feedback of reward or loss. ASL data revealed that MA reduced regional CBF in the ventral striatum and anterior insula. The drug produced its expected effects on subjective ratings, cardiovascular measures, and task-related reaction time. The current study extends previous findings (Knutson et al. 2004; Funayama et al. 2014) with a larger sample size (43 vs. 8) and adds to our understanding of the effects of the monoaminergic enhancing stimulant, MA, on processing positive and negative reward stimuli.

Our findings showed that MA increased ventral striatal and anterior insular activity during the anticipation of loss versus non-loss cues, consistent with the results of a previous study by Knutson et al. (2004). Knutson et al. found that a moderate dose of d-amphetamine increased activation in the nucleus accumbens during anticipation of both low and high magnitude loss, but decreased activation during anticipation of gains. Amphetamine also increased ratings of arousal in response to negative monetary cues, suggesting that they may have been more salient than reward anticipation cues. This supports the idea that the ventral striatum is involved in processing salient cues, regardless of valence (Haber and Knutson 2010; Liu et al. 2011; Miller et al. 2014; Oldham et al. 2018). Our findings from a larger sample size are broadly consistent with the Knutson findings, suggesting that stimulants enhance the salience of negative stimuli. It is also possible that the effects of MA on anticipation of loss were related to other behavioral actions of the drug indirectly related to reward. For example, there is evidence that the ventral striatum is activated during tasks that involve increased attentional demand (Elliott et al. 2004; Zink et al. 2004; Bjork and Hommer 2007; Breckel et al. 2011). The increase in ventral striatal activation after MA may reflect increased attentional processes and responsivity toward anticipatory loss versus non-loss cues that become more salient after drug induced increases in monoaminergic transmission.

In our current study, MA also increased activation of the anterior insula. The insula has been associated with motivational processes including the integration of subjective feelings with decisions for action (Namkung et al. 2017). It is also a major node within the salience network, which is involved in processing motivationally salient stimuli (Peters et al. 2016). Our finding that MA increased activation in the insula is consistent with the idea that stimulant drugs enhance the salience of motivational cues, in this case, cues predicting monetary loss. Unfortunately, in the present study, we did not record participants’ subjective responses to gains or losses during the task.

MA did not increase striatal activity during anticipation of gains versus non-gains. This finding is consistent with the report by Knutson et al. (2004), where amphetamine did not increase, and indeed decreased, activation in the striatum in response to gains. This effect may be the result of tonic increases at the synaptic level after amphetamines, which may decrease or mask stimulation-dependent dopamine release (Schmitz et al. 2001). In this way, MA may dampen responses to discrete, reward-related cues. Interestingly, Knutson et al. 2004 reported that d-amphetamine also tended to decrease subjective ratings of excitement after the presentation of high magnitude reward cues, further suggesting a dampening of responses to rewards.

Our ASL results showed that MA decreased CBF in the ventral striatum and the anterior insula. CBF is a measure of blood flow to the brain that is not representative of direct neural activity. Other studies have reported that MA decreases CBF (Polesskaya et al. 2011; Nordin et al. 2013). The findings that MA decreased CBF while increasing BOLD response during the anticipation of monetary loss are consistent with the idea that CBF and BOLD measure distinct neurovascular responses in response to MA. Indeed, we did not find a significant relationship between drug-induced ventral striatal BOLD activation during anticipation of loss versus neutral and change in ventral striatal CBF after MA. The BOLD response is thought to reflect the effect of MA on task-related brain activity.

The MID task is designed to assess neural activation during both anticipation and receipt of reward. In this study, MA altered neural responses during the anticipation phase, but not during receipt of either gains or losses. This is partly consistent with the findings of Knutson et al. (2004), who found that d-amphetamine also did not diminish neural activation during the receipt of loss, but in their study the drug increased activation in the mesial prefrontal cortex, posterior cingulate, and left nucleus accumbens during the receipt of monetary gain. This difference could be attributed to differing sample sizes between the studies. Together, these findings indicate that these moderate doses of amphetamines produce minimal effects on corticostriatal activity during receipt of monetary reward, in healthy participants.

This study had some limitations. First, although our sample was relatively large, the group was fairly homogeneous and consisted of young adults who had minimal lifetime history of prior drug use, and few psychiatric or medical conditions. Future studies should address this using a more heterogeneous population, or a clinically relevant population such as those with low response to reward (e.g. depression). Second, we combined low and high magnitude monetary incentive cues for all contrasts that were analyzed. By combining these, we did not examine potential magnitude-dependent effects. It is possible that larger magnitude cues would elicit greater neural activation. The effect of magnitude may also be detectable with a larger sample. Third, we did not examine changes in MA-induced brain activation during reward and loss processing in relation to subjective responses to the drug. Future studies can examine this with a larger sample to identify sources of individual variation in brain activation in response to rewards and losses.

The present study adds to our understanding of the effects of stimulants on reward and loss processing. Previous studies examining the effects of stimulants on the processing of reward and loss anticipation have found mixed results, perhaps due to low sample sizes. Here, we used a larger sample size (*n* = 43) and a within-subject design to help address this question. MA increased striatal and insular activation during anticipation of monetary loss, but did not affect activation during anticipation of gains, or during outcome feedback. These findings extend upon previous studies and help our understanding of MA’s effect on neural function. Our results suggest MA increases reactivity to the anticipation of monetary loss, perhaps because of actions on reward and attention-related circuits. This study may be used as a framework for understanding the effects of the drug in other populations, like those with depression or stimulant use disorders.

## Supplementary Material

Supplementary_materials_tgad014

## Data Availability

Data are available from the authors upon request.

## References

[ref1] Alsop DC, Detre JA, Golay X, Günther M, Hendrikse J, Hernandez-Garcia L, Lu H, MacIntosh BJ, Parkes LM, Smits M. Recommended implementation of arterial spin-labeled perfusion MRI for clinical applications: a consensus of the ISMRM perfusion study group and the European consortium for ASL in dementia. Magn Reson Med. 2015:73(1):102–116. 10.1002/mrm.25197. 24715426 PMC4190138

[ref2] Andersson JL, Skare S, Ashburner J. How to correct susceptibility distortions in spin-echo echo-planar images: application to diffusion tensor imaging. Neuroimage. 2003:20(2):870–888. 10.1016/S1053-8119(03)00336-7. 14568458

[ref3] Bernacer J, Corlett PR, Ramachandra P, McFarlane B, Turner DC, Clark L, Robbins TW, Fletcher PC, Murray GK. Methamphetamine-induced disruption of frontostriatal reward learning signals: relation to psychotic symptoms. Am J Psychiatry. 2013:170(11):1326–1334. 10.1176/appi.ajp.2013.12070978. 23732871

[ref4] Bjork JM, Hommer DW. Anticipating instrumentally obtained and passively-received rewards: a factorial fMRI investigation. Behav Brain Res. 2007:177(1):165–170. 10.1016/j.bbr.2006.10.034. 17140674 PMC1859851

[ref5] Breckel TP, Giessing C, Thiel CM. Impact of brain networks involved in vigilance on processing irrelevant visual motion. Neuroimage. 2011:55(4):1754–1762. 10.1016/j.neuroimage.2011.01.025. 21255659

[ref6] Chappell MA, Groves AR, Whitcher B, Woolrich MW. Variational Bayesian inference for a nonlinear forward model. IEEE Trans Signal Process. 2008:57(1):223–236.

[ref7] Cox RW . AFNI: software for analysis and visualization of functional magnetic resonance neuroimages. Comput Biomed Res. 1996:29(3):162–173. 10.1006/cbmr.1996.0014. 8812068

[ref8] Crane N, Molla H, de Wit H. Methamphetamine alters nucleus accumbens neural activation to monetary loss in healthy young adults. Psychopharmacology. 2023. 10.1007/s00213-023-06398-4. PMC10572040 37530883

[ref9] Cruickshank CC, Dyer KR. A review of the clinical pharmacology of methamphetamine. Addiction. 2009:104(7):1085–1099. 10.1111/j.1360-0443.2009.02564.x. 19426289

[ref10] Dugré JR, Dumais A, Bitar N, Potvin S. Loss anticipation and outcome during the *Monetary Incentive Delay Task*: a neuroimaging systematic review and meta-analysis. PeerJ. 2018:6:e4749. 10.7717/peerj.4749. 29761060 PMC5949205

[ref11] Elliott R, Newman JL, Longe OA, William Deakin JF. Instrumental responding for rewards is associated with enhanced neuronal response in subcortical reward systems. Neuroimage. 2004:21(3):984–990. 10.1016/j.neuroimage.2003.10.010. 15006665

[ref12] Fischman MW, Foltin RW. Utility of subjective-effects measurements in assessing abuse liability of drugs in humans. Br J Addict. 1991:86(12):1563–1570. 10.1111/j.1360-0443.1991.tb01749.x. 1786488

[ref13] Funayama T, Ikeda Y, Tateno A, Takahashi H, Okubo Y, Fukayama H, Suzuki H. Modafinil augments brain activation associated with reward anticipation in the nucleus accumbens. Psychopharmacology. 2014:231(16):3217–3228. 10.1007/s00213-014-3499-0. 24682502

[ref14] Haber SN, Knutson B. The reward circuit: linking primate anatomy and human imaging. Neuropsychopharmacology. 2010:35(1):4–26. 10.1038/npp.2009.129. 19812543 PMC3055449

[ref15] Izawa J, Yamanashi K, Asakura T, Misu Y, Goshima Y. Differential effects of methamphetamine and cocaine on behavior and extracellular levels of dopamine and 3,4-dihydroxyphenylalanine in the nucleus accumbens of conscious rats. Eur J Pharmacol. 2006:549(1–3):84–90. 10.1016/j.ejphar.2006.08.031. 16979160

[ref16] Jauhar S, Fortea L, Solanes A, Albajes-Eizagirre A, McKenna PJ, Radua J. Brain activations associated with anticipation and delivery of monetary reward: a systematic review and meta-analysis of fMRI studies. PLoS One. 2021:16(8):e0255292. 10.1371/journal.pone.0255292. 34351957 PMC8341642

[ref17] Knutson B, Greer SM. Anticipatory affect: neural correlates and consequences for choice. Philos Trans R Soc Lond Ser B Biol Sci. 2008:363(1511):3771–3786. 10.1098/rstb.2008.0155. 18829428 PMC2607363

[ref18] Knutson B, Bjork JM, Fong GW, Hommer D, Mattay VS, Weinberger DR. Amphetamine modulates human incentive processing. Neuron. 2004:43(2):261–269. 10.1016/j.neuron.2004.06.030. 15260961

[ref19] Knutson B, Westdorp A, Kaiser E, Hommer D. FMRI visualization of brain activity during a monetary incentive delay task. Neuroimage. 2000:12(1):20–27. 10.1006/nimg.2000.0593. 10875899

[ref20] Levy DJ, Glimcher PW. The root of all value: a neural common currency for choice. Curr Opin Neurobiol. 2012:22(6):1027–1038. 10.1016/j.conb.2012.06.001. 22766486 PMC4093837

[ref21] Liu X, Hairston J, Schrier M, Fan J. Common and distinct networks underlying reward valence and processing stages: a meta-analysis of functional neuroimaging studies. Neurosci Biobehav Rev. 2011:35(5):1219–1236. 10.1016/j.neubiorev.2010.12.012. 21185861 PMC3395003

[ref22] Luijten M, Schellekens AF, Kühn S, Machielse MW, Sescousse G. Disruption of reward processing in addiction: an image-based meta-analysis of functional magnetic resonance imaging studies. JAMA Psychiatry. 2017:74(4):387–398. 10.1001/jamapsychiatry.2016.3084. 28146248

[ref23] Lutz K, Widmer M. What can the monetary incentive delay task tell us about the neural processing of reward and punishment? Neurosci Neuroecon. 2014:3:33–45. 10.2147/NAN.S38864.

[ref24] Miller EM, Shankar MU, Knutson B, McClure SM. Dissociating motivation from reward in human striatal activity. J Cogn Neurosci. 2014:26(5):1075–1084. 10.1162/jocn_a_00535. 24345173 PMC5808846

[ref25] Morean ME, de Wit H, King AC, Sofuoglu M, Rueger SY, O’Malley SS. The drug effects questionnaire: psychometric support across three drug types. Psychopharmacology. 2013:227(1):177–192. 10.1007/s00213-012-2954-z. 23271193 PMC3624068

[ref26] Namkung H, Kim SH, Sawa A. The insula: an underestimated brain area in clinical neuroscience, psychiatry, and neurology. Trends Neurosci. 2017:40(4):200–207. 10.1016/j.tins.2017.02.002. 28314446 PMC5538352

[ref27] Nordin LE, Li TQ, Brogren J, Johansson P, Sjögren N, Hannesdottir K, Björk C, Segerdahl M, Wang DJ, Julin P. Cortical responses to amphetamine exposure studied by pCASL MRI and pharmacokinetic/pharmacodynamic dose modeling. Neuroimage. 2013:68:75–82. 10.1016/j.neuroimage.2012.11.035. 23246855

[ref28] O'Doherty JP . Reward representations and reward-related learning in the human brain: insights from neuroimaging. Curr Opin Neurobiol. 2004:14(6):769–776. 10.1016/j.conb.2004.10.016. 15582382

[ref29] Oldham S, Murawski C, Fornito A, Youssef G, Yücel M, Lorenzetti V. The anticipation and outcome phases of reward and loss processing: a neuroimaging meta-analysis of the monetary incentive delay task. Hum Brain Mapp. 2018:39(8):3398–3418. 10.1002/hbm.24184. 29696725 PMC6055646

[ref30] Peters SK, Dunlop K, Downar J. Cortico-striatal-thalamic loop circuits of the salience network: a central pathway in psychiatric disease and treatment. Front Syst Neurosci. 2016:10:104. 10.3389/fnsys.2016.00104. 28082874 PMC5187454

[ref31] Polesskaya O, Silva J, Sanfilippo C, Desrosiers T, Sun A, Shen J, Feng C, Polesskiy A, Deane R, Zlokovic B, et al. Methamphetamine causes sustained depression in cerebral blood flow. Brain Res. 2011:1373:91–100. 10.1016/j.brainres.2010.12.017. 21156163 PMC3026925

[ref32] Rothman RB, Baumann MH, Dersch CM, Romero DV, Rice KC, Carroll FI, Partilla JS. Amphetamine-type central nervous system stimulants release norepinephrine more potently than they release dopamine and serotonin. Synapse (New York, NY). 2001:39(1):32–41. 10.1002/1098-2396(20010101)39:1<32::AID-SYN5>3.0.CO;2-3. 11071707

[ref33] Schmitz Y, Lee CJ, Schmauss C, Gonon F, Sulzer D. Amphetamine distorts stimulation-dependent dopamine overflow: effects on D2 autoreceptors, transporters, and synaptic vesicle stores. J Neurosci. 2001:21(16):5916–5924. 10.1523/JNEUROSCI.21-16-05916.2001. 11487614 PMC6763160

[ref34] Schouw ML, de Ruiter MB, Kaag AM, van den Brink W, Lindauer RJ, Reneman L. Dopaminergic dysfunction in abstinent dexamphetamine users: results from a pharmacological fMRI study using a reward anticipation task and a methylphenidate challenge. Drug Alcohol Depend. 2013:130(1–3):52–60. 10.1016/j.drugalcdep.2012.10.010. 23142493

[ref35] Smith SM, Jenkinson M, Woolrich MW, Beckmann CF, Behrens TE, Johansen-Berg H, Bannister PR, de Luca M, Drobnjak I, Flitney DE, et al. Advances in functional and structural MR image analysis and implementation as FSL. NeuroImage. 2004:23(Suppl 1):S208–S219. 10.1016/j.neuroimage.2004.07.051. 15501092

[ref36] Völlm BA, de Araujo IE, Cowen PJ, Rolls ET, Kringelbach ML, Smith KA, Jezzard P, Heal RJ, Matthews PM. Methamphetamine activates reward circuitry in drug naïve human subjects. Neuropsychopharmacology. 2004:29(9):1715–1722. 10.1038/sj.npp.1300481. 15138439

[ref37] Weafer J, van Hedger K, Keedy SK, Nwaokolo N, de Wit H. Methamphetamine acutely alters frontostriatal resting state functional connectivity in healthy young adults. Addict Biol. 2020:25(3):e12775. 10.1111/adb.12775. 31099141 PMC6527344

[ref38] White TL, Justice AJ, de Wit H. Differential subjective effects of D-amphetamine by gender, hormone levels and menstrual cycle phase. Pharmacol Biochem Behav. 2002:73(4):729–741. 10.1016/s0091-3057(02)00818-3. 12213517

[ref39] Wilson RP, Colizzi M, Bossong MG, Allen P, Kempton M, MTAC, Bhattacharyya S. The neural substrate of reward anticipation in health: a meta-analysis of fMRI findings in the monetary incentive delay task. Neuropsychol Rev. 2018:28(4):496–506. 10.1007/s11065-018-9385-5. 30255220 PMC6327084

[ref40] Wu CC, Samanez-Larkin GR, Katovich K, Knutson B. Affective traits link to reliable neural markers of incentive anticipation. Neuroimage. 2014:84:279–289. 10.1016/j.neuroimage.2013.08.055. 24001457 PMC3849140

[ref41] Zhang Y, Loonam TM, Noailles PA, Angulo JA. Comparison of cocaine- and methamphetamine-evoked dopamine and glutamate overflow in somatodendritic and terminal field regions of the rat brain during acute, chronic, and early withdrawal conditions. Ann N Y Acad Sci. 2001:937:93–120. 10.1111/j.1749-6632.2001.tb03560.x. 11458542

[ref42] Zink CF, Pagnoni G, Martin-Skurski ME, Chappelow JC, Berns GS. Human striatal responses to monetary reward depend on saliency. Neuron. 2004:42(3):509–517. 10.1016/s0896-6273(04)00183-7. 15134646

