# Supplementary material for: Methamphetamine enhances neural activation during anticipation of loss in the monetary incentive delay task
Source: Cereb Cortex Commun. 2023 Jul 20;4(3):tgad014. doi: 10.1093/texcom/tgad014 (PMC10412021; doi:10.1093/texcom/tgad014)
Supplement: Supplementary_materials_tgad014 [file supplementary_materials_tgad014.docx]

**Supplementary Materials**

**Supp Table 1.** Adjusted *t*-value means (sem) for ROI’s during the anticipation and feedback phases of the MID

| **Anticipation Phase** | |  | | | |  | | | |  | | | |
| --- | --- | --- | --- | --- | --- | --- | --- | --- | --- | --- | --- | --- | --- |
|  | **Ventral striatum** | | | **Thalamus** | | | **Anterior Insula** | | | | **Caudate** | | |
|  | **placebo** | | **MA** | **placebo** | **MA** | | **placebo** | | **MA** | | **placebo** | | **MA** |
| *gain vs. non-gain* | 0.066 (0.07) | | 0.090 (0.06) | -0.087 (0.04) | -0.014 (0.03) | | 0.244 (0.07) | | 0.193 (0.06) | | -0.056 (0.09) | | 0.054 (0.1) |
| *loss vs. non-loss* | 0.001 (0.06) | | 0.237* (0.5) | -0.077 (0.03) | -0.042 (0.03) | | -0.051 (0.07) | | 0.153* (0.07) | | 0.160 (0.1) | | 0.234 (0.09) |
| *gain vs. loss* | 0.082 (0.06) | | -0.037 (0.05) | -0.011 (0.04) | -0.030 (0.03) | | 0.112 (0.07) | | 0.050 (0.06) | | -0.149 (0.07) | | -0.186 (0.07) |
| **Feedback (Reward Outcome) Phase** | | | | | | | |  | | | |  | |
|  | **Ventral Striatum** | | | **Amygdala** | | | **OFC/vmPFC** | | | | **PCC** | | |
|  | **placebo** | | **MA** | **placebo** | **MA** | | **placebo** | | **MA** | | **placebo** | | **MA** |
| *gain outcome* | 0.220 (0.07) | | 0.227 (0.06) | 0.286 (0.09) | 0.257 (0.08) | | 0.306 (0.1) | | 0.153 (0.08) | | 0.030 (0.1) | | 0.040 (0.1) |
| *loss outcome* | 0.093 (0.07) | | 0.183 (0.05) | 0.037 (0.1) | 0.110 (0.09) | | -0.058 (0.08) | | 0.031 (0.08) | | -0.076 (0.1) | | 0.177 (0.1) |

OFC: Orbitofrontal prefrontal cortex; vmPFC: ventromedial prefrontal cortex; PCC: posterior cingulate cortex
